# A scale space approach for unsupervised feature selection in mass spectra classification for ovarian cancer detection

**DOI:** 10.1186/1471-2105-10-S12-S9

**Published:** 2009-10-15

**Authors:** Michele Ceccarelli, Antonio d'Acierno, Angelo Facchiano

**Affiliations:** 1 Department of Biological and Environmental Sciences, University of Sannio, Via Port'Arsa 11, 82100, Benevento, Italy; 2 Bioinformatics Core, BIOGEM, Ariano Irpino, Italy; 3 Institute of Food Sciences, National Research Council, Via Roma 52 A/C, Avellino, Italy

## Abstract

**Background:**

Mass spectrometry spectra, widely used in proteomics studies as a screening tool for protein profiling and to detect discriminatory signals, are high dimensional data. A large number of local maxima (a.k.a. *peaks*) have to be analyzed as part of computational pipelines aimed at the realization of efficient predictive and screening protocols. With this kind of data dimensions and samples size the risk of over-fitting and selection bias is pervasive. Therefore the development of bio-informatics methods based on unsupervised feature extraction can lead to general tools which can be applied to several fields of predictive proteomics.

**Results:**

We propose a method for feature selection and extraction grounded on the theory of multi-scale spaces for high resolution spectra derived from analysis of serum. Then we use support vector machines for classification. In particular we use a database containing 216 samples spectra divided in 115 cancer and 91 control samples. The overall accuracy averaged over a large cross validation study is 98.18. The area under the ROC curve of the best selected model is 0.9962.

**Conclusion:**

We improved previous known results on the problem on the same data, with the advantage that the proposed method has an unsupervised feature selection phase. All the developed code, as MATLAB scripts, can be downloaded from

## Background

Proteomics studies are widely used in the biomedical research as an investigation tool to gain understanding of biological processes under specific conditions. Proteomics gives a detailed picture of the presence, integrity and/or modification of the whole mixture of proteins extracted by a source. For medical purposes, proteomics offers a diagnostic perspective for the early detection of pathologies as well as for the choice of the most effective therapy. In fact, samples as serum, plasma, and other kinds of extracts contain proteins for which the covalent structure may be modified by specific pathological states, which may induce or prevent processes as glycation, phosphorylation, methylation, or any other addition of a molecular group to the protein. As a more general case, a whole protein could be expressed or not under pathological conditions. In all these cases, the proteomics pattern analyzed by mass spectrometry techniques can evidence differences due to the pathology. Similarly, comparative proteomics can be exploited to evaluate the effects of a specific therapy.

Among the thousands of proteins and peptides present in a serum sample, which represent its proteome, few key signals may be significant markers of the pathological state, and their search within the proteome represents a still open field of research. The detection of the markers and their full characterization has a number of advantages, including the opportunity of using them for diagnostics uses and the improvement of the knowledge about the pathological effects at molecular level, required to develop new drugs and therapies.

Mass spectrometry [1] is the elective technique to characterize the proteome and its modification. The mass spectrum represents a molecular profile of the sample under analysis, obtained with increasing precision and automation techniques. Despite the large number of signals obtained in the proteome analysis, molecular modifications can be detected and markers of pathological states can be identified. MALDI-TOF (Matrix-Assisted Laser Desorption and Ionization Time-Of-Flight) is a common technology used in mass spectrometry, and SELDI-TOF (Surface-Enhanced Laser Desorption and Ionization Time-Of-Flight) is also used as a modified form of MALDI-TOF. According to these techniques, proteins are co-crystallized with UV-absorbing compounds, then a UV laser beam is used to vaporize the crystals, and ionized proteins are then accelerated in an electric field. The analysis is then completed by the TOF analyzer. Differences in the two technologies, which reside mainly in the sample preparation, make SELDI-TOF more reliable for biomarkers discovery and other proteomic studies in biomedicine.

Data produced by mass spectrometry are spectra, typically reported as vectors of data, describing the intensity of signals due to biomolecules with specific mass-to-charge (*m/z*) ratio values. Given the high dimensionality of spectra, given their different length and since they are often affected by errors and noise, preprocessing techniques are mandatory before any data analysis. After preprocessing (to correct noise and reduce dimensionality), several statistical and artificial intelligence based technologies could be used for mining these data.

A very important contribution of the application of mass spectrometry techniques to the classification of ovarian cancer is reported in [2] where the authors suggest a stochastic search method for selecting a subset of feature which best separates between healthy and pathological cases; the classification is based on a clustering approach using self-organizing maps and the authors show that the proposed method is able to classify all cancer cases and 95% of healthy women. The same methodology has been also successfully applied to high resolution spectrometry in [3] where the authors obtain a 100% sensitivity and specificity of classification over a random split (between training set and test set) of the data. The feature selection approach of both papers follows an optimization procedure having as objective function the discrimination ability of the adopted classifier over the subset of selected features. From the geometrical point of view it can be described as the selection of a random projection of data onto a subspace where the selected patterns are best separated. However, one should consider that in this kind of problems we have a small set of data (of order of some hundreds) in a very high dimensional space (of order of several thousands of points). Therefore, there is the risk that this good separation into the subspace could just be due to a random effect depending on the sparseness of the data [4]. In order to avoid this risk, large scale cross validation should be applied for the correct evaluation of the prediction accuracy [5].

Another important issue to be addressed in the selection of features for classification is the way of performing feature selection and cross validation together. In particular, if the feature selection step is external to the cross validation procedure, as for example in [6,7], *i.e *when the feature selection is done by using all the data and the performance evaluation by cross validation is performed just for the classification phase, then the obtained results may be severely biased due to the so called *selection bias *effect. An interesting experiment is reported in [8] where the selection bias effect produces perfect classification even for completely fake datasets. A more proper approach should validate by cross validation both classification algorithms and feature selection, and this can be easily done by leaving the test samples out of the dataset before undergoing feature selection [9]. One of the main contribution of the present paper is the validation of the dataset of [3] with a large scale cross validation study and the adoption of an unsupervised feature extraction showing that it is possible to classify the dataset with a very high accuracy without the selection bias effect.

The dataset adopted in the present work was also used in the paper [10] where the authors developed a preprocessing based on the Kolmogorov-Smirnov test, restriction of coefficient of variation and wavelet analysis. The classification step is then performed, as in our case, with support vector machines. Their method achieves an average sensitivity of 97.38% (sd = 0.0125) and an average specificity of 93.30% (sd = 0.0174) in 1000 independent *k*-fold cross-validations; here we have a better sensitivity and specificity as reported in the *Results*.

A study about the classification methods for ovarian cancer detection is reported in [11] where the authors compared two feature extraction algorithms together with several classification approaches on a MALDI TOF dataset. The *T*-statistic was used to rank features in terms of their relevance. Then two feature subsets were greedily selected (respectively having 15 and 25 features each). Support vector machines, random forests, linear/quadratic discriminant analysis (LDA/QDA), k-nearest neighbors, and bagged/boosted decision trees were subsequently used to classify the data. In addition, random forests were also used to select relevant features with previously mentioned algorithms used for classification. When the *T*-statistic was used as a feature extraction technique, support vector machines, LDA and random forests classifiers obtained the top three results (with accuracy in the vicinity of 85%). On the other hand, classification improved to approximately 92% when random forests were used as both feature extractors and classifiers. While these results appear promising, the authors provide little motivation as to why 15 and 25 feature sets were selected. Here we do not fix *a priori *the number of features letting the algorithm select automatically the number of features as function of the percentage of energy to be preserved in the PCA and the number of peaks in the analyzed average spectrum as reported below (*Methods*).

The PCA dimensionality reduction approach was also used in [12] with the dataset of [2] coupled with a nearest centroid classifier for classification. When training sets were larger than 75% of the total sample size, perfect (100%) accuracy was achieved on the OC-WCX2b data set. The author performed cross validation after feature selection, and as explained before, this results could be influenced by the selection bias effect. Using only 50% of data for training, the performance dropped by 0.01%. Unfortunately, the probabilistic approach used in the study can leave some samples unclassified. For the OC-H4 data set, the system had a 92.45% sensitivity and 91.95% specificity when 75% of the data was used for training with only 98.60% of the data samples classified.

## Results and discussion

The proposed feature extraction and classification method has been tested on a dataset available from the National Cancer Institute of the U.S. National Institute of Health consisting of 121 cancer samples and 95 control samples. Each sample is an high resolution spectrum with about 360000 points and m/z ranging from 700 to 12000. Some results on these data have been published in [3] and [10] and are useful for comparison with the method proposed here.

**Figure 1:**
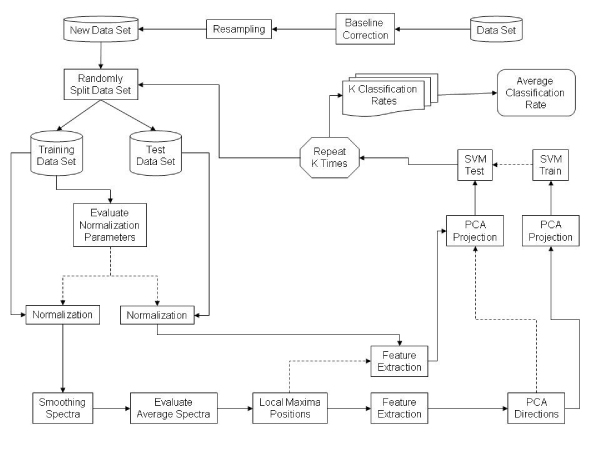
**The proposed method**. Dashed lines indicate control information.

In figure 1 the overall process used to test our solution is shown. After having independently corrected the baseline and re-sampled each spectrum, we started *k*-fold cross validation (we used *k *= 10). As it is well known, in *k*-fold cross validation the data set is randomly divided in *k *sets; of the *k *sets, a set is retained as the validation data for testing the model, and the remaining *k *- 1 subsamples are used as training data. The cross-validation process is repeated *k *times, with each of the *k *subsets of samples used exactly once as the validation data. The *k *results from the folds are then averaged to produce a single estimation. Using the training set we derive the normalization parameters that are used to normalize both the training and the test sets. The normalized training data set is then used for feature extraction (see *Methods*) obtaining the *m/z*'s of the peaks that best describe (according our method) each spectrum; these m/z's are then used to synthetically represent both the training spectra and the test spectra. Then, the training set is used to obtain PCA directions (obtained having fixed the overall energy); these directions are of course used to project both the training and the test sets. Last, the training set is used to train our SVM while the test set is clearly used to test the correct classification rate.

**Figure 2:**
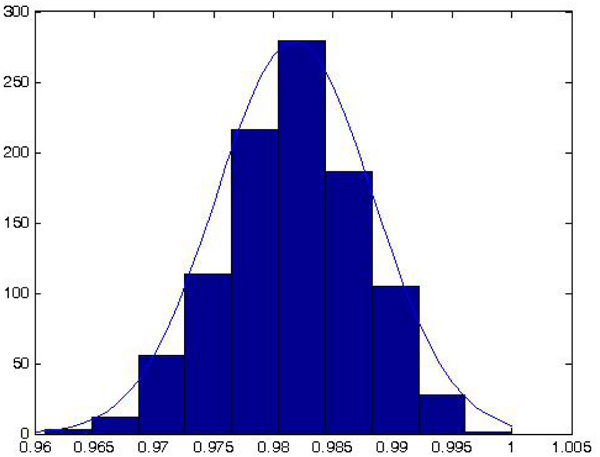
**The performance of the proposed system**.

In order to get the results, all the classification experiments and estimated classification rates are averaged over 1000 runs of the whole process. The main results are reported in figure 2: it contains the distribution of the accuracy of classification over each *k*-fold run on the test set and repeated 1000 times. The classification accuracies on these runs clusters around the average with a Gaussian shape having average 0.9818 and standard deviation 4.314 * 10^-5^. As we can notice, the classification accuracy is almost stable over the runs even if each run could eventually extract different peak sets to perform the classification. Moreover, we obtain a 100% accuracy in some of the runs, just as in [3], but without using peak selection based on the classification accuracy.

An interesting problem to be investigated is the *feature stability *of the feature extraction phase. Indeed, since for a correct cross validation procedure, the feature selection must be performed inside each fold, it is possible that different folds lead to different feature sets. Therefore the question whether there is a set of *stable features *which are maintained in different folds and runs arises. We run the whole feature extraction 1000 times using 10-folds and, at each iteration, we compute the intersection of the selected feature positions with the previous set. The final result being the set of selected peaks which are shared among all the 10000 runs.

**Figure 3:**
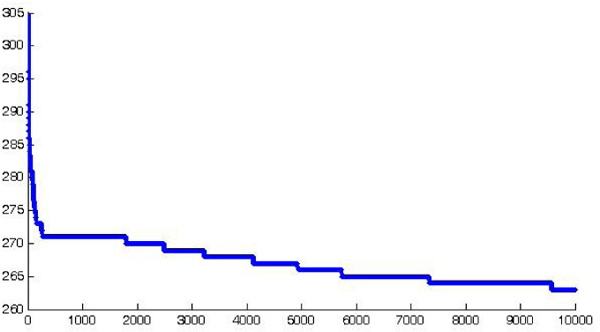
**The number of recurring features among different runs**.

We observe that the number of features rapidly decreases towards 265 (see figure 3) and that the algorithm tends to select repeatedly these core peaks. A further interesting observation concerns the fact that these stable features contain most of the peaks used by [3] in their work as reported in table 1. In particular, the results in the table show that the proposed method selects as stable features most of the values used by them and all the recurring *m/z*. Since our data are subject to a re-sampling step before feature selection, the matching is measured up to a small approximation error.

**Table 1:** The table reports the m/z values selected in [3] for the classification of the same dataset with four different models

**m/z features in **[3]	stable feature in our model
818.480	Yes
1001.654	Yes
1144.796	Yes
1255.593	Yes
1276.861	Yes
2374.244	No
4260.403	Yes
4292.900	Yes
4377.853	Yes
6004.416	Yes
6548.771	Yes
7046.018	Yes
**7060.121**	Yes
7096.922	No
7202.716	Yes
8540.536	Yes
**8605.678**	Yes
8664.385	No
**8706.065**	Yes
8709.548	Yes
9367.113	No
9870.937	No

The values in bold represent the features recurring between the various models reported by the authors. As it can be seen from the table, the proposed method selects as stable features most of the values and all the recurring *m/z*. Since our data are subject to a re-sampling step before feature selection, the matching is measured up to a small approximation error.

**Figure 4:**
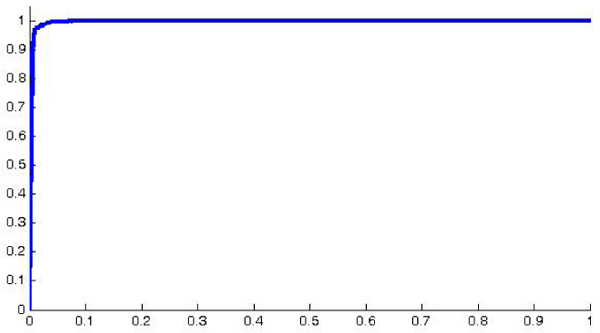
**The averaged ROC curve of 50 runs of the cross validation; the resulting AUC is 0.9962**.

Since we are facing a binary classification problem, it is significant to plot the false positive rate (*FPr*) versus the true positive rates (*TPr*) obtaining the receiver operating characteristics (ROC) curve. In order to obtain a ROC curve where all the samples are represented, we used a merging strategy of the test sets generated inside each fold. In this way we can plot a curve where each sample belongs to the test at least once. Moreover, since each run of the cross validation produces a curve, we used a vertical averaging approach to combine all the graphs [13]. This means that each curve is treated as a function *R *such that *R*(*FPr*) = *TPr *and assuming as averaged ROC curve (*FPr*) = *E *[*R*(*FPr*)] thus obtaining figure 4. The graph in figure 4 shows a curve representing the diagnostic ability of the classifier. In particular the area under the curve (AUC) of a binary classifier can be interpreted as the probability that the classifier will rank a randomly chosen positive instance higher than a randomly chosen negative instance. In our case, for the curve of figure 4, we obtained a high AUC of 0.9962.

Finally, if we want to consider the specificity and sensitivity of the classification method we obtained the results reported in table 2 as compared to that obtained in [10]. The table reports the averaged sensitivity and specificity over the 1000 run of the 10-fold cross validation.

**Table 2:** Accuracy of our system compared with the results reported in [10] on the same dataset.

	Proposed	[10]
Sensitivity	98.76%	97.38%
Specificity	98.48%	93.30%

### System tuning

As detailed in the section *Methods*, there are some basic parameters which can influence the whole performance of our approach:

• the maximum scale of signal smoothing, *σ*

• the peak averaging window size, (*WS*),

• the amount of energy, in percentage, retained in the Principal Component Analysis, *E*

• width of the kernel functions for the SVM classifier, *γ*;

It is important to evaluate the influence of the parameters and how the performance of the classification depends on them. We ran a large set of experiments for this purpose having:

• *σ *varying in the interval [0.05:1] using a step of 0.1;

• *WS *varying in the interval [1:5];

• *E *varying in the interval [0.70:0.98] using a step of 0.02;

• *γ *varying in the interval [2:8].

**Figure 5:**
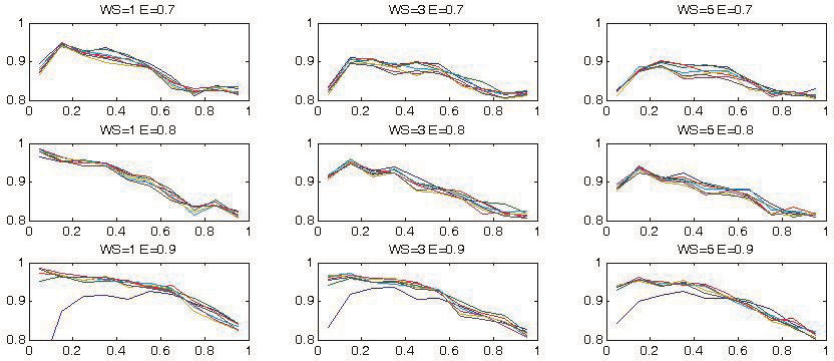
**The correct classification rate as a function of the scale, *σ*, having *γ *as parameter for different values of the energy (*E*) and of the windows size (*WS*)**.

**Figure 6:**
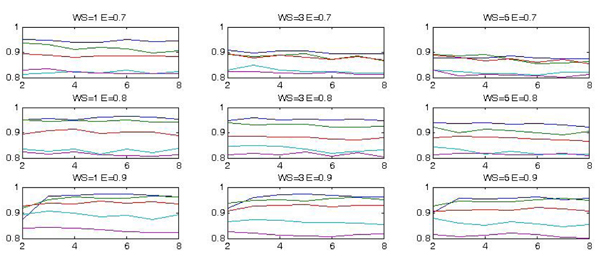
**The correct classification rate as a function of the *γ *having the scale as parameter for different values of the energy (*E*) and of the windows size (*WS*)**.

For each parameter configuration, *k*-fold (with *k *= 10) cross validation has been used to test the generalization performance. Each test has been repeated 5 times, so that each set has been tested 50 times: in this phase, the mean correct classification value has been used as quality measure. Figure 5 and figure 6 show the results we have obtained. In particular in the first figure we plot the various curves, representing the accuracy of classification in terms of *σ*, each curve in the plot is obtained by fixing *γ *and the various plots correspond to *WS *= 1, 3, 5 and *E *= 0.7, 0, 8, 0.9. The various curves follow almost similar shapes and there is not a well defined maximum; rather the optimum is obtained over a quite large interval of the considered values. The same is true if we consider the accuracy in terms of the SVM width *γ *as reported in figure 6.

## Conclusion

The paper presented the results obtained by applying a feature extraction procedure for mass spectra classification based on a scale-space analysis of the data. The features were then used to train a statistical classifier to discriminate between normal and cancer samples. In order to compare our results with state of the art methods, we adopted a public available dataset already analyzed by other researchers. We obtained an average accuracy of 98.18 of correct classification, 98.76% sensitivity and 98.48% specificity over a large cross validation experiment designed to be free of the selection bias effect: this improves previously known results [10]. We also analyzed how stable the feature selection methods is and showed that over a large set of runs the method tends to select the same set of features.

Another advantage of the adopted method consists in the use of the multi-scale properties of the spectra rather than a procedure based on the discrimination ability of the selected features. For the considered problem, with a high dimensional space and a small number of data points, optimal separating surfaces based on projection can be the results of chance rather than a subset of significant features. Finally, we used the discrimination accuracy as figure of merit just to compute the optimal parameters of the feature selection step.

## Methods

### Preprocessing

Before the feature selection phase, a preprocessing step is performed aimed to homogenization and correction of the spectra data. The spectral data produced by a single laser shot in a mass spectrometer consists of a vector of counts. Each count represents the number of ions hitting the detector during a small, fixed interval of time. A complete spectrum is acquired within tens of milliseconds, so a typical spectrum is a vector containing between 10,000 and 100,000 entries. Therefore, each spectrum contains many thousands of intensity measurements representing an unknown number of protein peaks, this requires extensive low-level processing in order to identify the locations of peaks. Inadequate or incorrect preprocessing methods, however, can result in data sets that exhibit substantial biases and make it difficult to reach meaningful biological results. In our experiments we applied the following preprocessing steps:

• *re-sampling*: Gaussian kernel reconstruction of the signal in order to have a set of *d*-dimensional vectors with equally spaced mass/charge values;

• *baseline correction*: removes systematic artifacts, usually attributed to clusters of ionized matrix molecules hitting the detector during early portions of the experiment, or to detector overload;

• *normalization*: corrects for differences in the total amount of protein desorbed and ionized from the sample plate;

All the details, adopted parameters and scripts of the preprocessing step can be downloaded from the accompanying web page containing all the data and code.

### Feature extraction

The feature selection and description is crucial for mass spectrometry since subsequent analyses are performed only on the selected features. Several methods have been proposed which often rely on biased data sets and can reach biological conclusion difficult to be interpreted [14,15].

Peak detection is the standard method for extracting features and several techniques to identify peaks among the background noise have been proposed (see for example [16]). Model based approaches have been adopted for the phase of feature selection of mass spectra data [17]. The model based methods typically perform a huge number of regressions to fit signal models to spectra. Here we adopt a hybrid method which is fast just as the peak selection methods, and at the same time tries to model the average spectrum at various scales. The basic principle adopted for our selection of features relies on the scale space theory of signal analysis [18,19]. The main idea of a scale-space representation is to generate a one-parameter family of derived signals in which the fine-scale information is successively suppressed. This principle preserves peaks or other feature to be artificially introduced through scales and forces the analysis to be from finer scale to coarser scales.

We thus assume that the peaks can be profitably used to describe the spectrum itself but, when two peaks are too close they should be considered as a single maximum. Therefore the multi-scale analysis can help in observing the same signal at coarser scales for feature detection and signal matching purposes, as the scale increases the signal becomes coarser. The scale space theory [18] is a framework particularly suited to approach problems dealing with signals containing features and details which cannot be described and measured prior of the observation. The main idea of the scale space theory is that if no prior information is available about what are the appropriate scales for a given data set, then the only reasonable approach for an analysis system is to represent the input data at multiple scales. This idea received different formalizations in literature. Maybe, the best way of describing the scale space theory is trough an axiomatic approach. Starting from a continuous signal *f*(*x*), a family of derived signal is generated, (*T*_*σ*_*f*)(*x*), depending on a scale parameter *σ *≥ 0 and (*T*_0_*f*)(*x*) = *f *(*x*); the (*T*_*σ*_*f*)(*x*) analysis represents a continuous smoothing process where as the scale parameter *σ *increases the data get more and more coarser. The basic property of every scale space analysis is the *causality *principle. Roughly speaking, the causality principle states that each feature at a coarse scale must have a cause at a finer scale. This means that the smoothing process does not introduce spurious features preserving peaks or other feature to be artificially introduced through scales and the analysis is forced to be from finer scale to coarser scales. Formally, it can be shown that every causal smoothing process must be governed by, or be the discretized version of, a parabolic partial differential equation obeying a maximum principle [20]. By combining causality with the notions of isotropy and homogeneity, which essentially mean that all spatial positions and all scale levels must be treated in a similar manner, Witkin [18] showed that the only linear scale-space representation obeying to the above principles must satisfy the diffusion equation, *i.e*. (*T*_*σ*_*f*) is solution of the following equation:

(1)

equivalently, in one dimensions, the smoothing process is described by a convolution with a Gaussian having variance *σ *:

(2)

**Figure 7:**
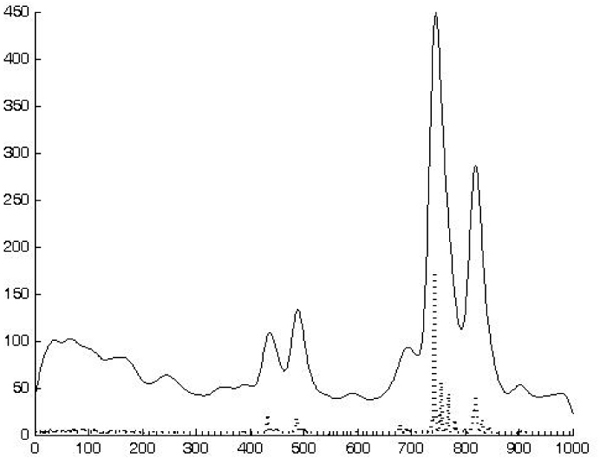
**A spectrum (dotted) end its smoothed version**.

The scale-space theory provides a well-founded framework for representing and detecting signal structures at multiple scales, however it does not address the problem of how to select locally appropriate scales for further analysis. Whereas the problem of finding the best scales for handling a given real-world data set may be regarded as intractable unless further information is available, some approaches have been proposed, for example [19] selects the scales at which normalized measures of feature strength assume local maxima with respect to scale. For the purposes of our approach we select the best scale *σ *by cross validation as described in *System Tuning*. As can be seen in figure 7, near peaks collapses in a single local maximum.

#### Peak selection and reduction

**Figure 8:**
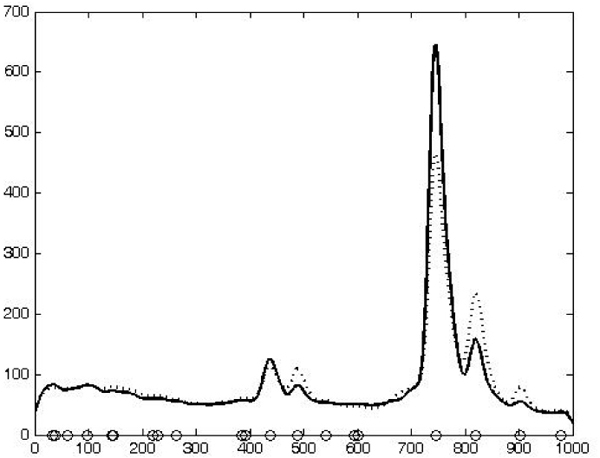
**The curve obtained summing smoothed spectra of cancer data (dotted) and the one one obtained summing the healthy data**. Circles on the *m/z *axis represent local maxima points.

In order to select a set of significant peaks to be considered as features, we use the local maxima of the average spectrum *E *[*T*_*σ*_*f *(*x*)]. These local maxima are considered as the locations of the considered peaks, see figure 8. Finally, each spectrum will be described by the mean value assumed by the original spectrum in a window centered in each of the selected local maxima.

**Figure 9:**
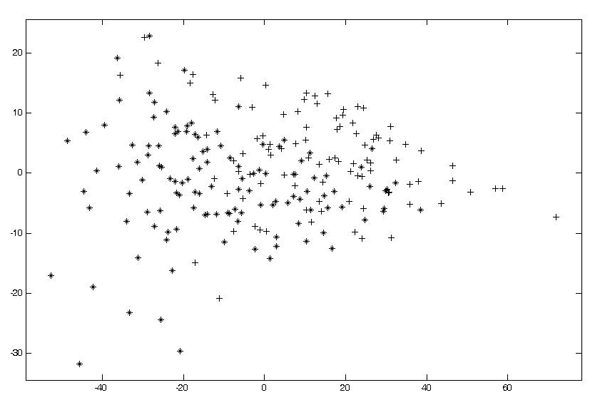
**The data we have to classify: the first component against the second one**.

As a last feature extraction step we perform a principal component analysis (PCA) for dimensionality reduction of the data collected as the intensity values over the selected windowed peak positions (see figure 9). It is important to point out that the feature selection must be performed inside the cross validation in order to not incur into the selection bias effect. In particular, all the parameters such as mean multi-scale spectrum, peak locations and principal components are computed inside the folding, this guarantees that the all the feature selection and extraction procedure is completely blind with respect to the test data. Another important point to underline is that the feature extraction and selection described so far does not use discrimination as a measure to identify useful peaks for the classification of data. This means that the proposed feature selection method is completely unsupervised and as such it is unbiased. Even with this simplification the classification accuracy both in terms of sensitivity and specificity and AUC beats other methods such as [10] over the same dataset.

### Classification

The classification problem can be naturally cast into the theory and practice of disciplines as Pattern Recognition and Machine Learning [21]. Support Vector Machine (SVM) is a technique [22] for Pattern Recognition and Data Mining classification tasks. While at present there exists no general theory that guarantees good generalization performances of SVM, but only probability bounds on its performance accuracy, there is a growing interest in this technique due to a wide literature reporting good performances in various heterogeneous fields [23].

In the case of linearly separable patterns on two-classes vectors it is straightforward to show the basic ideas of SVM: given a set of points in ℜ^*k *^and a two-class labels vector, SVM aims to find a linear surface that splits them in two groups according to the indicated labels, in the best possible way. Intuitively, if data are linearly separable (that is if it exists *at least *one hyperplane that splits them in two group), the problem becomes how to define and how to find *the best *possible hyperplane to do it. The SVM answer is that the best possible hyperplane is the one that maximizes the *margin*, that is the one that has maximal distance from *both *sets of points.

To generalize further, it is possible to consider surfaces that are not linear and work on a different model. By using a kernel function all data can be projected onto another space (possibly with infinite dimension) where they are linearly separable and perform the classification linearly in this new space. In practice if we look at the underlying optimization problem, it is easy to see that data appear only in the form of dot products and hence data transformed through a function *ϕ *appear also in the form *K*(*x*_*i*_, *x*_*j*_) = *ϕ *(*x*_*i*_) · *ϕ *(*x*_*j*_). Such a dot product function is called *Kernel*. Whatever function that satisfies dot product's constraints can be used as *Kernel *function, and there is an active field of research in the choice of the most suitable kernel for a given problem [24]. In this work Kernel's choice was derived form general practical considerations [25,26]:

• the Radial Basis Function (RBF) SVM has infinite *capacity *and hence gaussian RBF SVM of sufficiently small width can classify an arbitrarily large number of training points correctly;

• the RBF kernel includes as a special case the linear kernel;

• the RBF kernel behaves like the sigmoid kernel for certain parameters' values;

• the RBF kernel has less hyper-parameters than the polynomial kernel;

• the RBF kernel has less numerical difficulties than other kernels.

## Competing interests

The authors declare that they have no competing interests.

## Authors' contributions

MC designed the procedure, AdA designed and implemented the software and performed the experiments, AF proposed the biological problem and discussed the results. All authors contributed to the design of the whole work and to the writing of the manuscript. All authors read and approved the final manuscript.
